# Prosthetic Rehabilitation of a Partially Dentate Patient With a Maxillary Cast Partial Denture and Mandibular Overdenture: A Case Report

**DOI:** 10.7759/cureus.28652

**Published:** 2022-08-31

**Authors:** Akansha V Bansod, Sweta G Pisulkar, Seema Sathe, Arushi Beri, Chinmayee Dahihandekar

**Affiliations:** 1 Department of Prosthodontics, Crown & Bridge, Sharad Pawar Dental College and Hospital, Datta Meghe Institute of Medical Sciences, Wardha, IND

**Keywords:** cross arch stabilization, stability, cast partial denture, overdenture, residual ridge resorption

## Abstract

Using natural teeth as denture abutments can significantly slow the progression of residual ridge resorption. The abutments and the denture-bearing areas can share the stress concentration. By providing sensory feedback, occlusal stability loss, aesthetic loss, and compromised mastication, overdentures can help reduce residual ridge resorption. Overdentures have been shown to be effective in reducing residual ridge resorption and increasing retention and stability. When edentulous areas are too large or numerous for the fixed prosthesis and cross-arch stabilization is required, a cast partial denture (CPD) is preferred. The insertion and removal of the denture, as well as regular oral hygiene, are simple procedures. The current case report describes the prosthetic rehabilitation of a partially dentate patient using a maxillary CPD and mandibular overdenture.

## Introduction

Edentulousness is a condition in which tooth loss causes a negative aesthetic and biomechanical squeal. Although there has been a decrease in the number of complete denture patients, partially edentulous patients have increased in number, possibly due to global aging as well as oral health-related prevention policies. Prosthetics offer a variety of options for rehabilitating missing teeth in partially edentulous patients, including removable partial dentures (RPDs), and partial dentures, that are either tooth supported or implant supported [1].

The number and location of missing teeth have a large impact on how well the prosthesis restores and maintains functions similar to natural dentition [2]. Cast partial dentures (CPDs) produce improved results in terms of retention, stability, comfort, masticatory efficiency, and health of the periodontium of the abutment teeth. Conventional treatment of complete dentures can sometimes result in poor retention, stability, and satisfaction of the patient. In such cases, the patient's confidence and comfort are jeopardized [3]. Removable partial dentures, such as CPDs, are becoming obsolete as a treatment option for patients who are able to receive a fixed prosthesis. Regardless of whether a fixed prosthesis is not indicated, they remain the treatment of choice, particularly in medically compromised patients as in the mentioned case.

Overdentures, on the other hand, may be able to compensate for the limitations of the conventional complete denture [4]. Overdentures can solve problems like loosening of dentures, loss of proprioception, and residual ridge resorption, and thus they are the treatment option that tries to maintain the natural teeth and helps patients avoid becoming edentulous [5]. Despite recent developments in dental implantology, the conservative approach to root preservation followed by an overdenture is still valid. The treatment plan chosen is determined by the surrounding teeth and tissues, as well as the needs of the patient and financial status. Here we describe a case of Kennedy’s Class I cast partial denture in the maxillary arch and a tooth-supported overdenture in the inframaxillary arch in the mandibular arch.

## Case presentation

A 67-year-old male patient presented to the prosthodontics department with a chief complaint of missing teeth and difficulty eating. His medical history revealed that he had been hypertensive for seven years and was taking an antihypertensive medication orally. The patient stated that he had a dental extraction done about two months ago in the maxillary right and the left posterior region. The patient did not want to undergo any extensive treatment or surgical procedure, and hence, a conservative treatment had to be planned.

**Figure 1:**
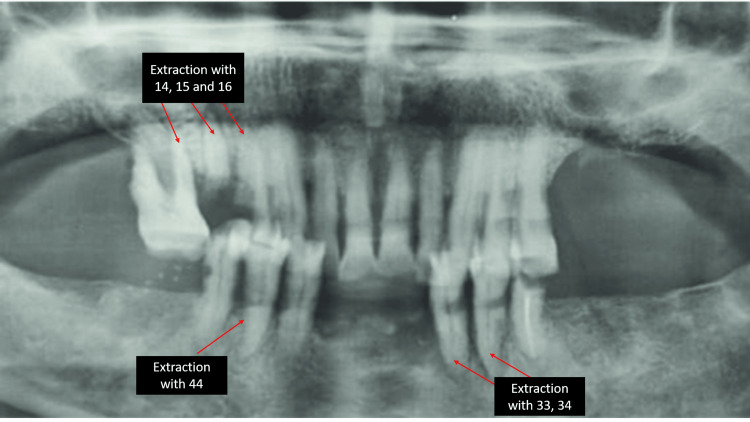
Radiographic examination Maxillary arch: extractions with 14, 15 and 16 Mandibular arch: extractions with 33, 34 and 44

In the preliminary inspection, it was found that the patient was missing teeth 17, 18, 26, 27, 28, 31, 32, 36, 37, 38, 41, 42, 46, 47 and 48, and had root pieces with 15 and grossly carious 14. Oral hygiene was adequate. A clinical examination correlated with the radiographic examination. The orthopantomograph revealed a poor prognosis with 14, 15, 16, 33, 34 and 44 and the patient was advised to undergo extractions (Figure 1).

**Figure 2:**
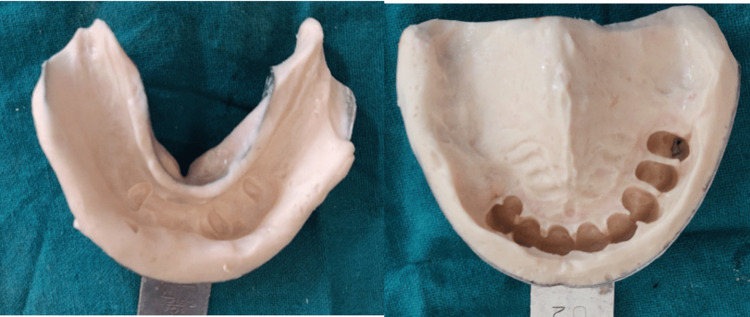
Maxillary and mandibular diagnostic impressions made with alginate

Following the mouth preparation phase, diagnostic impressions were made in an irreversible hydrocolloid impression alginate material (Zhermack Tropicalgin; Zhermack, Italy); diagnostic models were poured into the dental stone (Zhermack Elite Model Stone) and a bite registration of the present occlusion was made (Figure 2). The impressions were poured, and the diagnostic models were placed on a mean value articulator. The model was surveyed for diagnostic purposes.

**Figure 3:**
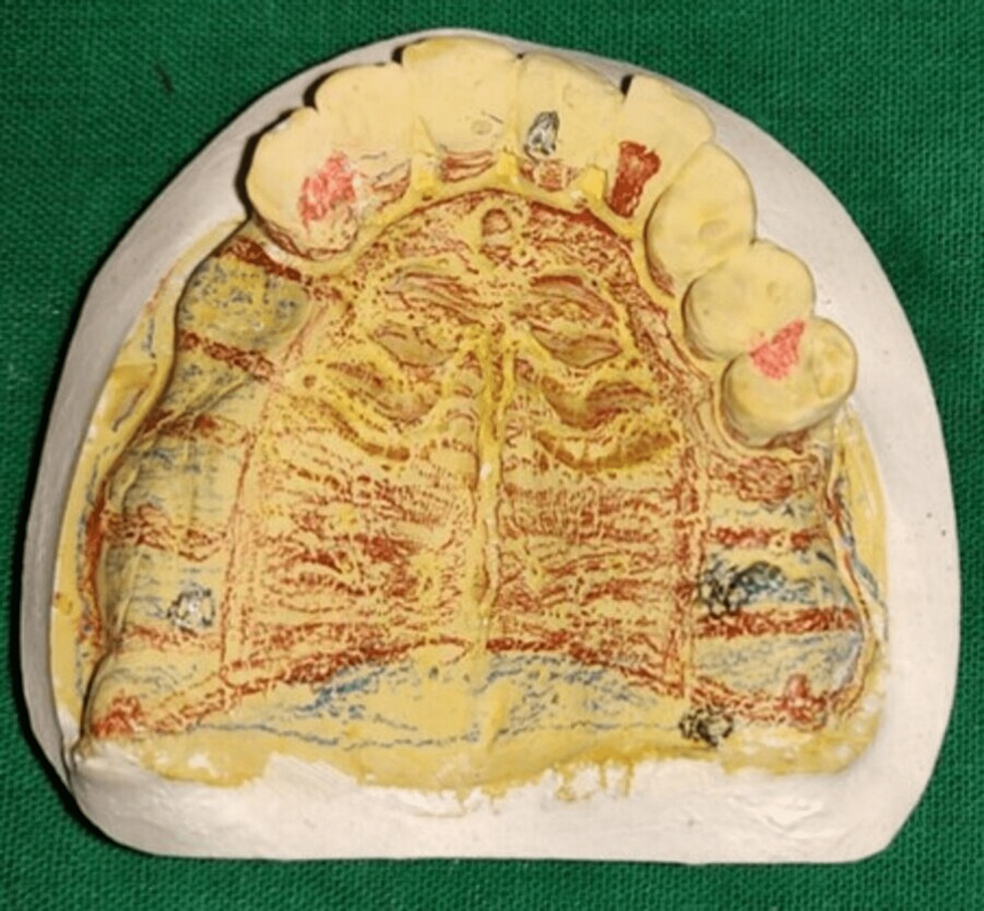
Design of the cast partial denture on the maxillary cast

The patient was prosthetically rehabilitated with a tooth-supported overdenture for the mandibular arch and maxillary arch, with a Kennedy Class I cast partial denture (Figure 3).

**Figure 4:**
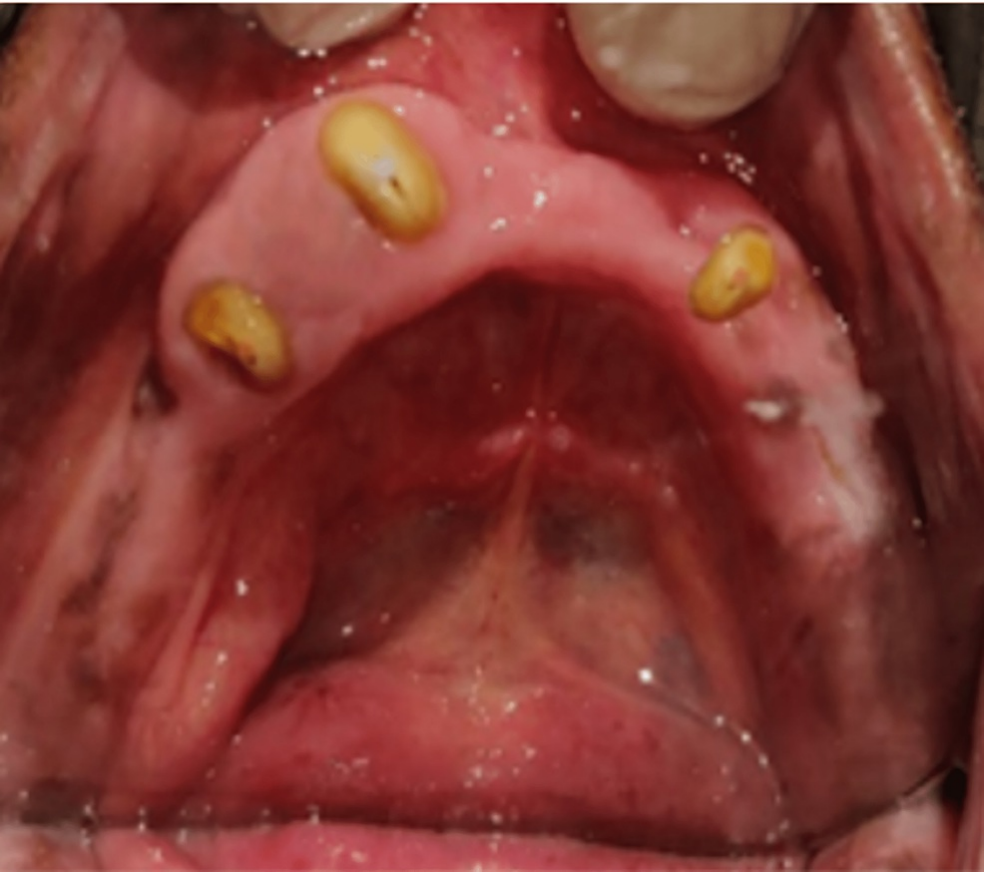
Tooth preparation for metal copings for the tooth-supported overdenture (mandibular)

Intentional root canal treatment (RCT) was carried out on 43 and 45. They were prepared with tapered, round-end diamond points with chamfer finish lines made subgingivally after RCTs with respect to 35, 43 and 45. Teeth were prepared dome-shaped in all dimensions, with an approximately 3-4 mm height, prominent just above the gingiva (Figure 4).

**Figure 5:**
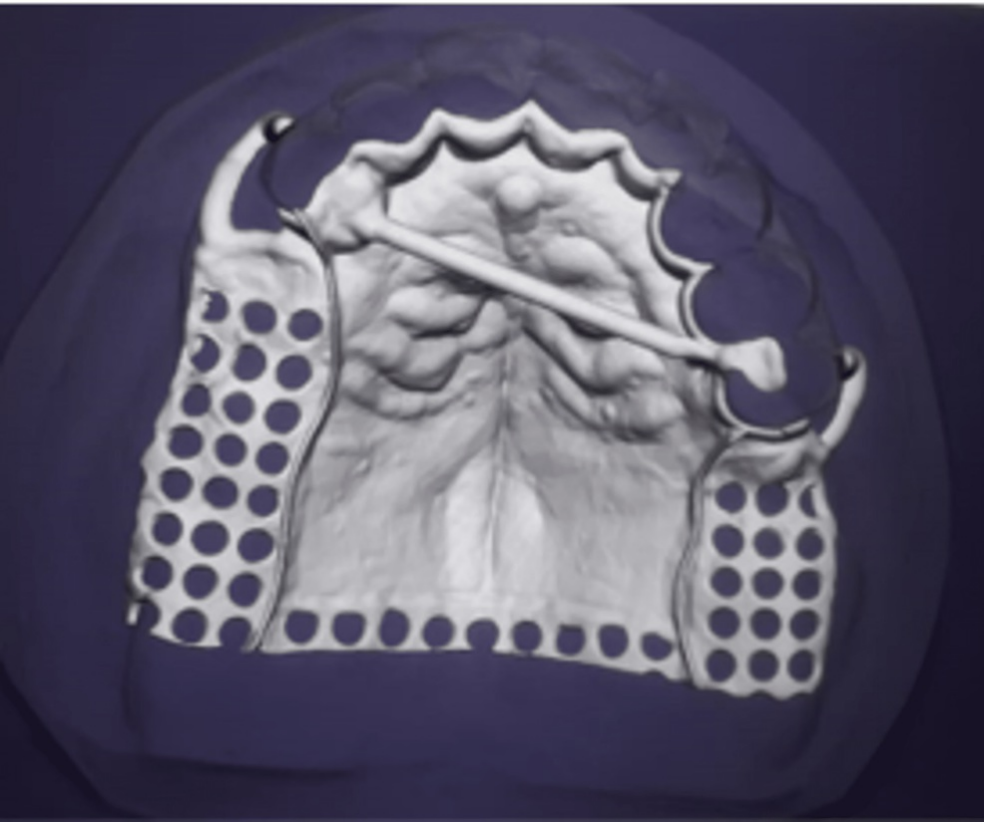
Designing of the cast partial denture using digital exocad software (maxilla) exocad software; exocad GmbH, Germany

Following the mandibular arch gingival retraction, a final impression was made with additional silicone using the two-step impression technique. The impression was used to create the master model, which was then used to make the copings. In the maxillary arch, surveying was done and the design of the cast partial denture was finalized on the digital scan (Figure 5).

**Figure 6:**
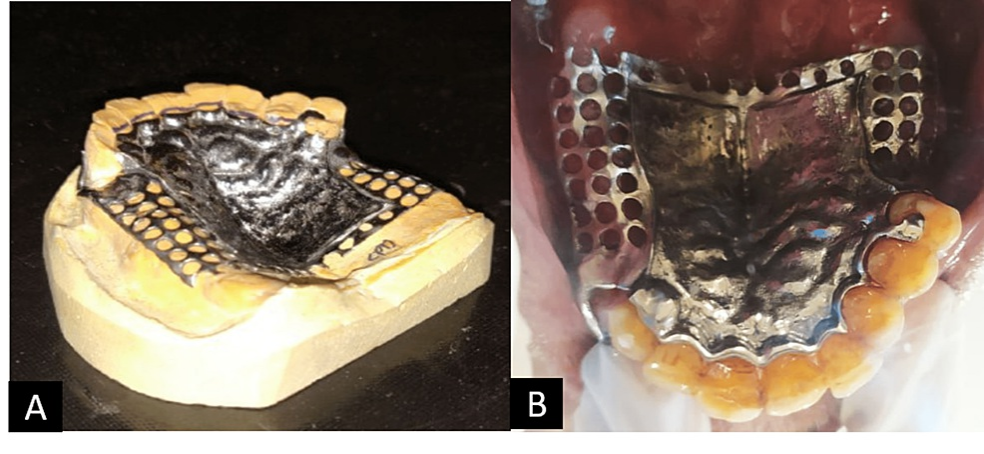
Metal framework try-in (A) on cast and (B) intraorally

**Figure 7:**
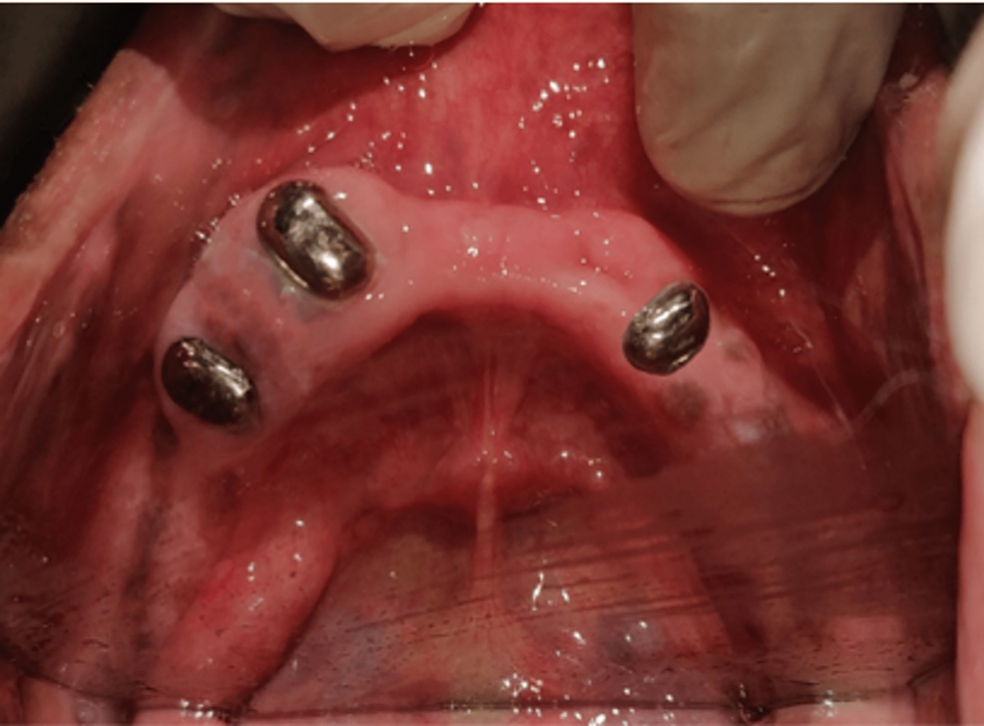
Metal copings cemented on the mandibular arch

Rest seats were prepared on teeth 13 and 25, guiding planes were prepared and final impressions were recorded. The final impression was created with the heavy- and light-body addition silicone impression material and the master cast was poured; the agar hydrocolloid was used for duplication. The refractory cast was created using the material for investment. A partial cast design was created and fabricated, and a wax pattern was created on the refractory cast. The casting of the framework was completed and alloy Co-Cr was used in the casting. The framework was reduced in size as well as polished and adjusted over the master cast (Figure 6). Copings were cemented on the mandibular arch (Figure 7).

**Figure 8:**
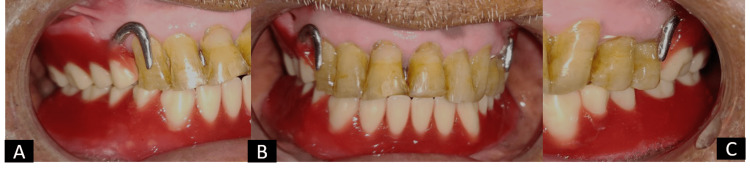
Try-in procedure: (A) right lateral view, (B) frontal view and (C) left lateral view

**Figure 9:**
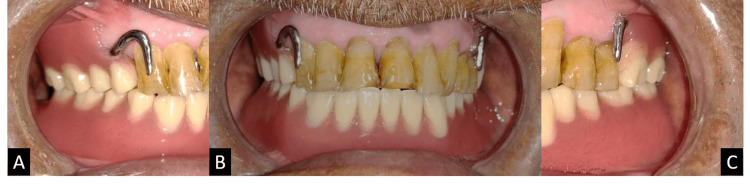
Final prosthesis insertion: (A) right lateral view, (B) frontal view and (C) left lateral view

After assessing the framework, jaw relation was recorded in the mouth with a wax rim and mounted on a semi-adjustable articulator, followed by a try-in procedure (Figure 8). Figure 9 shows the prostheses in situ.

## Discussion

A maxillary cast partial denture and a mandibular overdenture were the choices of treatment for this patient because of their excellent retention and stability; also, they provide stress distribution and good proprioception by the remaining natural teeth. Alternative options for treatment consisted of extraction of other remaining teeth, followed by a traditional complete denture therapy. This was not chosen because teeth extraction would have reduced proprioception and available support provided by the natural teeth and the periodontium that supports them. An implant-supported prosthesis was not selected as the treatment option as the patient was medically compromised, and also, not willing to undergo any surgical procedure [6-9].

However, the limitations of removable maxillary cast partial dentures include patient adaptation problems and the necessity of some time for the patient to adapt. RPDs can also cause plaque build-up around abutment teeth, which can lead to carious lesions and periodontal diseases. Some improperly designed RPDs may exhibit aesthetic issues as a result of metal display. The disadvantages of tooth-supported overdentures include the need for meticulous oral hygiene to prevent caries and periodontal disease. The overdenture is typically larger and more contoured that may cause encroachment of the inter-occlusal distance.

For a patient, the outlook of losing all the teeth is very upsetting. It also lowers a patient's morale because it serves as a reminder of being dependent and senescence indirectly. In such cases, overdentures as a preventive prosthodontic treatment modality should be implemented on a regular basis in our dental clinics due to their numerous benefits. In a five-year study, Crum and Rooney graphically demonstrated an average loss of 0.6 mm of vertical bone in the anterior part of the mandible of overdenture patients via cephalometric radiographs, compared to a 5.2 mm loss in complete denture patients [10]. In 1978, Rissin et al. compared masticatory performance in patients with natural dentition, complete denture and overdenture. They found that masticatory efficiency with overdentures was better than that with complete dentures [11]. In a research, it was concluded that the presence of removable partial dentures is important for dietary intake and that replacing missing teeth may help people maintain a healthy diet [7]. It has been found that partial tooth loss affects food acceptability in the same way as edentulism is associated with a poor diet and compromised nutrition, and tooth loss may cause dietary changes. As a result, removable treatment options are preferred in patients who are unable to receive fixed prostheses. Furthermore, the presence of teeth preserves proprioception in overdenture patients, improving patient acceptability and satisfaction. Hence, the current case report justifies the successful rehabilitation of a partially dentate mouth using a maxillary cast partial denture and mandibular overdenture.

## Conclusions

The restoration of a partially edentulous mouth without compromising the needs of the patient poses challenges in decision-making when it comes to treatment planning. The technique used for our patient was a simple one but provided a treatment plan providing the best possible care for an individual. The maxillary cast partial denture served a better prosthesis as stable, functional and biological restoration, whereas the tooth-supported mandibular overdenture provided proprioception and improved masticatory efficiency over complete dentures.

## References

[1] Kukunoor S, Savadi RC, Venkata Krishnam Raju K, Kumar S (2014). A viable treatment alternative in distal extension cases: a case report. J Indian Prosthodont Soc.

[2] Eggbeer D, Bibb R, William R (2005). The computer aided design and rapid prototyping fabrication of removable denture frameworks. J Eng Med.

[3] Thakare KS, Bhongade ML, Charde P, Kale S, Jaiswal P, Somnath BK, Pendor S (2013). Genetic mapping in Papillon-Lefèvre syndrome: a report of two cases. Case Rep Dent.

[4] Godbole S, Phakhan AJ, Kale S, Dahane T (2016). Prosthodontic onsiderations of speech in complete denture. IOSR J Dent Med Sci.

[5] Mistry R, Pisulkar SK, Borle AB, Godbole S, Mandhane R (2018). Stability in complete dentures: an overview. IOSR J Dent Med Sci.

[6] Balwani TR, Dubey SG, Sathe S, Chandak A (2020). Demystifying role of phonetics in complete denture. Int J Res Pharm Sci.

[7] Watson CL, Reeve PE, Barnes E, Lane AE, Bates JF (1986). The role of personality in the management of partial dentures. J Oral Rehabil.

[8] Runov J, Kroone H, Stoltze K, Maeda T, El Ghamrawy E, Brill N (1980). Host response to two different designs of minor connector. J Oral Rehabil.

[9] Al-Jammali ZM, Saad Z, Saad M (2020). Prosthodontics clinical cases (patients with decreased inter-arch distance-case report). IJMRA.

[10] Crum RJ, Rooney GE Jr (1978). Alveolar bone loss in overdentures: a 5-year study. J Prosthet Dent.

[11] Rissin L, House JE, Manly RS, Kapur KK (1978). Clinical comparison of masticatory performance and electromyographic activity of patients with complete dentures, overdentures, and natural teeth. J Prosthet Dent.

